# Laparoscopic Hepatectomy for Colorectal Liver Metastases: The Current State of the Art

**DOI:** 10.3389/fonc.2019.00442

**Published:** 2019-05-31

**Authors:** Aali J. Sheen, Saurabh Jamdar, Ajith K. Siriwardena

**Affiliations:** ^1^Regional Hepato-Pancreato-Biliary Unit, Manchester Royal Infirmary, Manchester, United Kingdom; ^2^Biomedicine Research Centre, Manchester Metropolitan University, Manchester, United Kingdom; ^3^Faculty of Biology, Medicine and Health, University of Manchester, Manchester, United Kingdom

**Keywords:** laparoscopic, hepatectomy, colorectal liver metastases (CLM), liver–surgery, minimally invasive

## Abstract

Hepatectomy together with systemic chemotherapy is the treatment of choice for patients with liver-limited colorectal metastases. Although the open approach to hepatectomy remains a standard option, there is increasing recognition of the potential advantages of laparoscopic hepatectomy. Laparoscopic approaches have become standardized and are the subject of two international consensus conferences. Major laparoscopic hepatectomy is currently being evaluated in international multi-center trials. The available data to date would indicate that there is oncological equivalence between open and laparoscopic approaches but that the latter is associated with less post-operative pain, shorter hospital stay and an earlier recovery of full function. Surgeons embarking on this approach must be experienced both in the techniques of advanced liver surgery and in laparoscopic surgery.

Hepatic resection together with systemic chemotherapy has become the backbone of treatment for patients with liver-limited colorectal metastases (1). Over the last decades of the twentieth century, hepatic resection for colorectal liver metastases became more widely available and was associated with low operative mortality as a result of improvements in anesthetic and operative technique together with better peri-operative care (2–4). Nevertheless, open hepatectomy remains a major undertaking. Patients require invasive monitoring and typically have epidural catheters for post-operative analgesia. Surgical access to the liver for open hepatectomy involves a lengthy subcostal or bi-subcostal incision and fixed costal margin retraction. This incision is required regardless of whether a major or minor liver resection is undertaken. In turn, recovery from this surgical wound is a major feature of the post-operative period and beyond. In the early post-operative period pulmonary complications could be related to the combination of limitation of chest wall excursion as a result of intra-operative fixed costal margin retraction and a painful upper abdominal incision. Although enhanced recovery protocols are widely implemented in modern liver surgery programmes, the loss of mobility after open liver surgery may contribute to the increased risk of deep vein thrombosis and pulmonary embolism (5, 6). Post-operative liver impairment after major open hepatectomy leading to hypoalbuminaemia could contribute to wound breakdown and dehiscence (7). In the longer term, there is a risk of incisional hernia (8).

Laparoscopic gastrointestinal surgery is now widely accepted as the standard of care for procedures such as cholecystectomy with more complex procedures such as colectomy and gastrectomy also being routinely undertaken by minimal access approaches (9–11).

Thus, it is logical that the laparoscopic approach would be applied to hepatectomy. Laparoscopic liver resection has the attraction of avoiding the morbidity associated with a lengthy incision. The main reservations expressed about the adoption of the laparoscopic approach for liver surgery were related to the ability to control intra-operative hemorrhage, the risk of air embolus and whether these approaches were oncologically equivalent to their open counterparts.

The first minimal access procedure to be established in the treatment of colorectal hepatic metastases was laparoscopic left lateral sectionectomy (left hepatic lobectomy). Laparoscopically this operation follows the same procedural steps as its open counterpart. Parenchymal transection is relatively minimal and the use of vascular staplers facilitates control of left lobe inflow and outflow. This relative ease and safety of adoption led to at least one expert surgical group declaring that laparoscopy should be the standard approach for patients requiring left hepatic lobectomy and the early conclusion of the ORANGE study comparing laparoscopic to open left lateral sectionectomy on grounds of poor accrual (12, 13). However, patients with liver metastases confined to segments II and III constitute only a minority of individuals with liver involvement by metastatic colorectal cancer. The laparoscopic approach was also utilized for patients requiring resections of the readily accessible lower/anterior segments: IVb, V, and VI. The first international consensus conference on laparoscopic liver surgery in Louisville, Kentucky stated in 2008 that “currently acceptable indications for laparoscopic liver resection are patients with solitary lesions, 5 cm or less, located in liver segments 2–6. The laparoscopic approach to left lateral sectionectomy should be considered standard practice” (14). The continued professional focus on laparoscopic liver surgery for colorectal hepatic metastases led to a second consensus conference at Morioka in Japan (15). This consensus conference emphasized the need for structured training before surgeons embark on laparoscopic major hepatectomy.

In terms of enhancing the body of evidence for laparoscopic hepatectomy for colorectal liver metastases after the Morioka consensus OSLO-COMET is a landmark study (16, 17). In this study 280 patients with resectable liver metastases from colorectal cancer were randomly assigned to undergo laparoscopic (*n* = 133) or open (*n* = 147) parenchyma-sparing liver resection. The postoperative complication rate was 19% in the laparoscopic surgery group and 31% in the open-surgery group (12% points difference [95% confidence interval 1.67–21.8; *P* = 0.021]). The postoperative hospital stay was shorter for laparoscopic surgery (53 vs. 96 h, *P* < 0.001), whereas there were no differences in blood loss, operation time or in clear resection margins. Mortality at 90 days did not differ significantly between the laparoscopic (0 patients) and open groups (1 patient). The authors concluded that for patients undergoing parenchyma-sparing liver resection for colorectal hepatic metastases, laparoscopic surgery was associated with significantly fewer postoperative complications compared to open surgery (16). The conclusions of OSLO-COMET are supported by the findings of a systematic review of the published literature of laparoscopic liver resection which reported outcome in 9,527 procedures (18). This review reported 37 deaths (mortality rate 0.4%) and in comparison to open surgery there were fewer complications, less blood loss, and a shorter hospital stay.

At this stage in the evolution of laparoscopic hepatectomy, evidence is still lacking on the safety of laparoscopic major hepatectomy for colorectal hepatic metastases and also on the feasibility of the laparoscopic approach for tumors in the right posterior sector and/or the segments close to the hepatic venous outflow (VIII and IVa). Two large, international, multi-center studies ORANGE II Plus (laparoscopic compared to open right hepatectomy), and ORANGE segments (laparoscopic compared to open hepatectomy for tumors involving any of segments VII, VIII, and/or IVa) are due to report soon and may influence the treatment landscape (19, 20). The current role of laparoscopic liver surgery is also well-summarized in detail in the Southampton consensus guidelines (21).

So, what then is the current state of the art as applied to laparoscopic hepatectomy for colorectal liver metastases? In overview, disease staging and assessment of patients for surgery is as it was for open surgery. Peri-operative preparation is simpler than in patients undergoing open surgery as the need for epidural analgesia can be avoided and thus patients can be mobilized immediately after surgery. There is evidence of less post-operative pain, shorter hospital stay, and earlier return to full function together with oncological equivalence. The rationale underlying hepatic resection itself has also evolved from an era where classical anatomical segmental hepatectomy was favored to the current understanding that the cancer biology of metastases is arguably best served by a parenchyma-preserving resection, ensuring complete resection (R0). Laparoscopic hepatectomy should maintain this approach.

Laparoscopic hepatectomy utilizes the principles established in the open hepatectomy era: liver mobilization and control of inflow and outflow are key components of safe laparoscopic liver surgery. In terms of mobilization of the liver, use of the left lateral decubitus position facilitates mobilization of the right lobe from the vena cava under direct vision. If required, the hepatocaval ligament can be dissected and divided (typically with a vascular stapler) and the right hepatic vein can be controlled extra-hepatically. An intracorporeal “snugger” can be safely placed around the structures of the hepatoduodenal ligament to facilitate the Pringle maneuver. Given the loss of tactile feedback, laparoscopic intra-operative ultrasonography remains critically important for confirming the location of lesions, excluding disease in the future remnant liver and in identifying inflow/outflow structures in the planned transection line.

Hepatic parenchymal transection at laparoscopic hepatectomy can be undertaken using the ultrasonic suction aspirator (CUSA excel, Valleylab, Boulder, Colorado) or using an energy device coupled with vascular staplers. This approach permits control of the major hepatic veins within the liver parenchyma. Pneumoperitoneum may facilitate haemostasis. It is important to assess the transection surface without pneumoperitoneum at the end of transection.

Thus, it can be said that laparoscopic hepatectomy is now part of the treatment portfolio for patients with colorectal hepatic metastases. Other technical evolutions of hepatic surgery for colorectal liver metastases such as associating ligation of the portal vein with liver resection (ALPPS) remain to be fully evaluated in the laparoscopic context (22).

Important questions remain about training in laparoscopic hepatectomy. In this context it is noteworthy that the OSLO-COMET authors had undertaken some 400 laparoscopic liver resections before embarking on their study. Thus, laparoscopic major hepatectomy requires the experienced liver anesthetic and surgical skills of the open era but also demands additional facility with laparoscopic mobilization, parenchymal transection, and haemostasis.

Looking to the future, it seems likely that the advantages in tissue handling in minimal access surgery seen with robotic approaches may make this the technique of choice for laparoscopic major hepatectomy.

In summary laparoscopic hepatectomy for colorectal hepatic metastases is a mainstream treatment. The available data to date would indicate that there is oncological equivalence between open and laparoscopic approaches but that the latter is associated with less post-operative pain, shorter hospital stay, and an earlier recovery of full function. Surgeons embarking on this approach must be experienced both in the techniques of advanced liver surgery and in laparoscopic surgery. Although it is likely that both patient and clinician preference will drive continued development in laparoscopic surgery at the present time questions remain about the benefits of this technique when applied to major right-sided hepatectomy and also to liver resection for posterior/superior hepatic segments.

## Author Contributions

AJS, SJ, and AKS were all involved in the design and structure of the manuscript. AKS formulated the idea and cited the majority of the references.

### Conflict of Interest Statement

AJS, SJ, and AKS have received hospitality from BOWA (Tubingen, Germany). AJS serves as a consultant to Medtronic and Baxter UK. The reviewers AG-W and ZS and the handling Editor declared their shared affiliation.
